# The Role of Hypertension in Cognitive Dysfunction

**DOI:** 10.3390/jcm13195979

**Published:** 2024-10-08

**Authors:** Panagiotis Theofilis, Georgia Doumani, Georgia-Christina Tsatsani, Nikolaos Volis, Aikaterini Kampourelli, Vasileios Thimis, Eleni Xanthopoulou, Rigas Kalaitzidis

**Affiliations:** Hypertension Excellence Center, Center for Nephrology “G. Papadakis”, General Hospital of Nikea-Piraeus “Ag. Panteleimon”, 184 54 Nikea, Greece; panos.theofilis@hotmail.com (P.T.); geo.doumani@gmail.com (G.D.); georgiachtsatsani@gmail.com (G.-C.T.); volnikvas@yahoo.gr (N.V.); kab.katerina@hotmail.com (A.K.); vthimis@gmail.com (V.T.); exanthopoulou8@gmail.com (E.X.)

**Keywords:** hypertension, cognitive function, cognitive impairment, dementia, antihypertensive therapy

## Abstract

Cognitive impairment and subsequent dementia are considered significant health challenges. In patients with established dementia, it is argued that hypertension is the main risk factor for small vessel ischemic disease and additional cortical white matter lesions. Cognitive domains and impairments associated with hypertension include learning, memory, attention, abstract reasoning, mental flexibility, psychomotor skills, and executive function. It is uncontrolled hypertension in midlife—but not late life—that is associated with worse cognitive impairment. Advanced imaging techniques confirm the effect of uncontrolled hypertension in developing dementia. Functional changes in the arterial system and an increase in arterial stiffness could be involved in the onset of dementia. In most studies, it is argued that better blood pressure control and duration of antihypertensive medication are associated with the incidence of dementia. In this review, the available data on the relationship between cognitive dysfunction and hypertension are examined.

## 1. Introduction

Cognitive impairment and subsequent dementia are considered significant health challenges [1]. Cognitive dysfunction refers to attention deficit, verbal and non-verbal education, short-term and working memory, visual and acoustic processing, problem solving, speed of processing, and motor function of patients. Furthermore, cognitive dysfunction could be the main mediator of a decline towards a major depressive disorder [2]. 

In the latest guidelines on the management of arterial hypertension, it is supported that uncontrolled hypertension in middle age could affect the incidence of cognitive dysfunction, Alzheimer’s disease and, vascular dementia [3]. Midlife is the transitional period of life between young adulthood and old age generally acknowledged as one’s early 40s to early 60s, with increases in longevity and health. However, blood pressure (BP) levels are not considered a major risk factor for cognitive decline, as opposed to the risk of developing cardiovascular or kidney disease [4]. In the most recent guidelines of the American Heart Association/American College of Cardiology for the management of hypertension, this is mentioned in seven published studies that assessed the association between BP reduction and cognitive function (with the exclusion of dementia), with only two supporting the prevention of cognitive decline with lowering blood pressure [4].

In patients with diagnosed dementia, hypertension is supported as the main risk factor for ischemic small vessel disease and additional white matter damage in the brain’s cortex, including in patients with Alzheimer’s disease. In most observational studies, improved control of systolic BP (SBP) could reduce the incidence of Alzheimer’s disease and other forms of dementia [5]. Data are substantial for BP reduction in middle age in comparison to elderly hypertensive patients. In European guidelines, it is supported that BP reduction could prevent cognitive decline with an uncertain action, however [3]. Moreover, the choice of antihypertensive therapy could have a limited impact, especially in cases where SBP is not adequately controlled. Several studies have shown that stringent SBP control (<130 mmHg) could delay the development of cortical white matter lesions and limit cognitive function decline [3]. This review examines the existing data on the relationship between cognitive dysfunction and hypertension. 

## 2. The Role of Hypertension in Cognitive Function

It is believed that over 9.5 million cases of dementia are attributed to uncontrolled hypertension worldwide [5]. Therefore, assessment of cognitive function and decline should be routinely included in the examination of hypertensive individuals above 65 years of age [6]. Hypertension can lead to impairments across various cognitive areas, such as learning, memory, attention, abstract reasoning, mental flexibility, psychomotor abilities, and executive function [7]. Of these, executive function, motor neuron activity, and attention are the most frequently affected cognitive domains in those with hypertension [8,9].

Hypertensive cerebrovascular disease causes damage to the prefrontal cortex, making it difficult to formulate goals, causing abstraction, difficulty in initiation, planning, organizing, and sequencing thoughts [7,10]. Uncontrolled hypertension is generally associated with various conditions of cognitive impairment, including sudden cognitive decline, poor cognitive performance, mild cognitive impairment, and dementia [11].

## 3. Association of Hypertension with Cognitive Dysfunction: Clinical Studies

For decades, numerous studies have associated uncontrolled hypertension with cognitive decline. The Syst-Eur study, a double-blind placebo-controlled trial, included elderly patients with elevated SBP and investigated whether antihypertensive treatment could reduce the incidence of dementia [12]. This study showed that aggressive treatment with a calcium channel blocker, nitrendipine, halved the rate of dementia from 7.7 to 3.8 cases per 1000 patient years [12]. 

In a longitudinal population-based study of elderly individuals in western France, researchers examined whether initial high BP and the administration of antihypertensive treatment predicted the onset of cognitive decline [13]. Based on the final assessment at 4 years, the risk of cognitive decline was estimated to be 4.3 (95% confidence interval (CI), 2.1 to 8.8) for those not receiving antihypertensive treatment and 1.9 (95% CI, 0.8 to 4.4) for those receiving treatment. Additionally, among individuals with high BP, cognitive decline appeared relatively quickly, and the risk was higher in hypertensive patients who did not receive treatment [13].

In a recent meta-analysis of five randomized studies involving 28,008 patients [14], the effect of antihypertensive treatment on dementia was evaluated. Over a follow-up period of 4.3 years, antihypertensive treatment was found to reduce the risk of dementia by 13% with a reduction in systolic/diastolic BP by 10/4 mmHg [14].

## 4. Target Blood Pressure and Cognitive Function

The optimal levels of BP for the reduction in the incidence of cognitive decline have been a matter of debate [15]. In a prospective longitudinal epidemiological study on aging conducted in Honolulu (The Honolulu Asia Aging Study (HAAS)), which examined risk factors and neuropathological disorders associated with cognitive decline and dementia in elderly Japanese–American men, untreated high BP in midlife was found to be a significant risk factor for the development of dementia [16]. The data showed that 27% of dementia cases could be attributed to SBP levels greater than 120 mmHg among men who had not received adequate treatment [16].

Additionally, in a randomized study conducted in the United States and Puerto Rico among hypertensive individuals aged 50 and older without a history of diabetes or stroke [17], the impact of intensive BP control on the risk of developing dementia was evaluated. Participants were randomized to either a target SBP level of <120 mmHg (intensive treatment group, n = 4678) or a target SBP level of <140 mmHg (standard treatment group, n = 4683). Among the 9361 participants (mean age, 67.9 years, 3332 women (35.6%)), 8563 (91.5%) completed at least one cognitive function assessment. The median intervention period was 3.34 years. Intensive BP control significantly reduced the risk of mild cognitive impairment (14.6 vs. 18.3 cases per 1000 person–years; HR, 0.81; 95% CI, 0.69–0.95). Additionally, intensive BP control significantly reduced the combined rate of mild cognitive impairment or dementia (2204 cases of dementia per 1000 person–years; HR, 0.85; 95% CI, 0.74–0.97). However, in this study, treatment aiming for SBP levels < 120 mmHg compared to <140 mmHg did not lead to a significant reduction in the risk of probable dementia. Due to the early termination of the study and the lower-than-expected incidence of dementia, it was considered that the study might have been underpowered for this specific endpoint [17]. 

Ιt is of great interest that individual studies in elderly patients support a U-shaped relationship between BP levels and cognitive function [18,19]. An analysis including data from the Rotterdam Study and the H-70 Study in Gothenburg [18] indicated that hypertension in older ages seemed to protect against future dementia. Specifically, the relationship between BP levels and dementia risk was examined, with consideration of potential modification by age, in a dataset from these two prospective studies. Participants were sourced from the Rotterdam Study (n = 6668), a longitudinal population-based study of individuals aged 55 years and older, and the H-70 Study in Gothenburg (n = 317), a study of individuals aged 85 years at baseline. The relative risk of dementia was estimated using Cox proportional hazards regression adjusted for age, sex, and location. The median follow-up was estimated at 2.1 years. Based on the results, the risk of dementia decreased with increasing BP levels (for every 10 mmHg increase in SBP, RR = 0.93, 95% CI = 0.88–0.99; and for every 10 mmHg increase in diastolic BP (DBP), RR = 0.89, 95% CI = 0.79–1.00). This association was observed primarily in individuals using antihypertensive medications. Individuals with dementia at study onset showed a greater reduction in BP levels during follow-up compared to those without dementia. This study clearly indicates an inverse relationship between BP levels and the risk of dementia in elderly individuals receiving antihypertensive treatment. These patients may potentially require higher BP levels to maintain adequate cerebral perfusion [19]. Finally, in an analysis of data from the prospective Rotterdam Study, which included 3078 men and women aged 55 to 84 years, along with 276 men and women aged 85 years and older from the prospective Leiden 85-plus Study [19], it was found that among younger participants (age < 65 years), both systolic and DBP were not associated with cognitive function 11 years later. However, for individuals aged 65 to 74 years, higher initial systolic and DBP values were associated with worse cognitive function 11 years later. In contrast, among older individuals (≥75 years), higher systolic and DBP levels were associated with better cognitive function at the end of the follow-up period. This effect was more pronounced in the oldest age group (age > 85 years). Thus, in this study, increased levels of BP were associated with a greater risk of cognitive decline in individuals younger than 75 years old but with better cognitive function in those older than 75 years old [19]. The authors concluded that in the elderly, lower levels of BP could lead to cerebral hypoperfusion and, ultimately, impaired cognitive function [19].

## 5. The Role of Diastolic Blood Pressure in Cognitive Function

The available data regarding the association between DBP levels and cognitive function come from a recent post hoc analysis of the SPRINT MIND study (Systolic Blood Pressure Intervention Trial Memory and Cognition in Decreased Hypertension) [20]. Participants were randomly assigned to either an intensive target (SBP < 120 mmHg; n = 4278) or a standard target (SBP < 140 mmHg; n = 4385). In the same population, cognitive function and cerebral blood flow were evaluated based on quartiles of DBP levels.

It was found that patients in the intensive BP control group had lower rates of dementia or mild cognitive impairment compared to the standard group, regardless of the quartiles of DBP. The hazard ratio for possible dementia or cognitive impairment in the intensive control group compared to the standard target was 0.91 (95% CI, 0.73–1.12) in the lowest quartile of DBP and 0.70 (95% CI, 0.48–1.02) in the highest quartile of DBP, respectively, with an interaction *p*-value of 0.24. Similar results were observed for possible dementia (interaction *p* = 0.06) and mild cognitive impairment (interaction *p* = 0.80).

Additionally, the effect of intensive therapy on cerebral blood flow was not affected by initial DBP levels (interaction *p* = 0.25). Finally, among participants within the lowest quartile of DBP, intensive compared to standard BP treatment led to a trend towards increased cerebral blood flow annually (+0.26 (95% CI, −0.72 to 1.24) mL/(100 g.min)). Therefore, it appears that intensive BP control did not have detrimental effects on cognitive function and cerebral blood flow in patients with low initial DBP levels [20].

## 6. Age-Related Risk of Cognitive Decline

The effect of BP levels on cognitive function in advanced age appears stronger when considering BP levels in midlife. A characteristic example comes from cohort analysis within the ARIC study [9], where untreated hypertension and increased SBP in midlife are associated with worse cognitive dysfunction over a 20-year follow-up period. In this study, greater cognitive decline was observed in white participants compared to African American participants at higher SBP levels during midlife [9]. These findings suggest that racial differences may exist in how hypertension impacts cognitive function during midlife. Additionally, it highlights the importance of considering racial and demographic factors when studying the effects of hypertension on cognition. It should be noted that cognitive decline was confirmed over the 20-year follow-up period across three repeated cognitive tests, and these associations were not detected in individuals with increased BP levels in their later years [9]. 

In another case–control study that included six European electronic health record databases, there were 291,780 cases of dementia compared to 29,170,549 controls. It was found that body mass index (BMI), SBP, and total cholesterol levels were lower in dementia cases compared to the control group at the time of diagnosis [21]. Additionally, in a cohort of 4.28 million individuals without known vascular disease and dementia identified in the Clinical Practice Research Datalink in the UK, SBP levels over time were associated with vascular dementia at the time of diagnosis [22]. Moreover, associations between SBP levels and dementia were identified in a population cohort with transient ischemic attack and stroke (Oxford Vascular Study). The association between usual SBP and the risk of dementia decreased with age (hazard ratio 1.62, 95% CI 1.13–2.35 per 20 mmHg higher SBP at ages 30–50 years; 1.26, CI 1.18–1.35 at ages 51–70; and 0.97, CI 0.92–1.03 at ages 71–90; *p* trend = 0.006). In the population-based cohort, SBP was predictive of a 5-year dementia risk without evidence of a negative correlation at older ages, regardless of prior transient ischemic attack or stroke [22].

## 7. The Role of Imaging in Hypertension-Associated Dementia

Today, advanced imaging techniques confirm the significant importance and impact of untreated hypertension in the development of dementia [23]. Studies utilizing imaging techniques to assess cognitive function and dementia underscore the importance of untreated hypertension in midlife [23]. Elevated BP precedes damage to cerebral white matter and suggests that hypertension treatment in the general population could mitigate the progression of white matter lesions [23].

People with uncontrolled untreated hypertension demonstrate significantly greater progression in cerebral white matter lesions compared to hypertensive individuals receiving treatment (difference (95% CI), 0.12 (0.00–0.23) mL/year) [23], supporting the view that elevated BP precedes white matter damage and that hypertension treatment could reduce the progression of white matter lesions in the general population. Together with the National Heart, Lung, and Blood Institute Twin Study [24], there is evidence that elevated BP during midlife was associated not only with a greater number of white matter lesions later in life but also with smaller brain parenchymal volume. 

Based on these studies, it is evident that elevated BP in midlife is a significant prognostic factor for both future cognitive dysfunction and structural brain changes demonstrated through magnetic resonance imaging in the later years of life. Additionally, since reduced neurobehavioral function correlates with decreased brain volume and increased white matter lesions, it can be inferred that the long-term impact of elevated BP on late-life cognitive decline may be due to its chronic, detrimental effects on brain structural characteristics [24].

In Uppsala, Sweden, a population-based cohort study of 999 men followed from age 50 [25] showed that those exhibiting a “non-dipping” circadian pattern of BP and persistently high 24 h BP, as assessed by ambulatory BP monitoring at age 70, also demonstrated poorer cognitive function. These findings further support the belief in early intervention in BP regulation to prevent cognitive decline and dementia [25].

In the Framingham Offspring Cohort study, involving 549 participants aged 55 to 64.9 years without a history of stroke or dementia, followed for 5 to 7 years [26], neurocognitive assessments and brain magnetic resonance imaging (subsample, n = 454) evaluated interactions between hypertension and the APOE-epsilon gene. In the total sample, elevated pulse pressure was associated with worse executive function, smaller brain volume, and larger ventricular volume. Among carriers of the APOE-epsilon4 gene, pulse pressure was also correlated with longitudinal decline in visual–spatial organization. These findings suggest that the appearance of increased arterial stiffness, indicated by elevated pulse pressure, may play a significant role in early cognitive decline and brain atrophy from midlife to late life, particularly among carriers of the APOE-epsilon4 gene [26]. 

Another component of BP that is of interest is its variability, which may be a significant risk factor for reduced cognitive function. Higher variability in BP from visit to visit, independent of mean arterial pressure, was associated with diminished cognitive function in old age [27].

## 8. The Role of Arterial Stiffness in Cognitive Impairment of Hypertensive Patients

Evidence continues to mount supporting the correlation between increased arterial stiffness and cognitive dysfunction [28,29]. This association was assessed in 308 elderly individuals reporting memory impairment [28]. A significant correlation was observed between pulse wave velocity (PWV) and cognitive function (*p* < 0.0001). PWV is a measure of arterial stiffness and reflects the speed at which pressure waves travel through the arteries. It is an important indicator of cardiovascular health; higher PWV values suggest increased stiffness, which can be associated with higher risks of cardiovascular events. In this study [28], PWV was significantly higher in individuals with vascular dementia (15.2 ± 3.9 m/s) or Alzheimer’s disease (13.3 ± 2.9 m/s) compared to those without cognitive decline (11.5 ± 2.0 m/s, *p* < 0.001). Additionally, PWV was higher in individuals with cognitive impairment (12.6 ± 2.6 m/s) compared to those without cognitive decline (11.5 ± 2.0 m/s, *p* = 0.01). These results highlighted a significant correlation between indices of arterial stiffness and cognitive dysfunction, suggesting that functional changes in the arterial system may ultimately contribute to the onset of dementia (vascular or Alzheimer’s type) [28].

In a single-center, cross-sectional observational study, a group of 600 elderly patients with memory loss was compared to 55 age-matched controls without dementia [29]. Brachial–ankle pulse wave velocity (baPWV) was higher in Alzheimer’s disease, vascular dementia, and mixed dementia compared to mild cognitive impairment and the control group (*p* < 0.05). BaPWV extends the PWV measurement by assessing arterial stiffness in both the upper and lower extremities. It calculates the velocity of pressure waves between the brachial artery and the ankle, providing a more comprehensive evaluation of vascular health. Elevated baPWV values can indicate generalized arterial stiffness and an increased risk of cardiovascular issues. Higher baPWV predicted the presence of Alzheimer’s disease, vascular dementia, mixed dementia, and mild cognitive impairment with odds ratios of 6.46, 8.74, 6.16, and 6.19, respectively [29]. Findings from this study also indicate that patients with mild cognitive impairment and dementia have stiffer arteries compared to the control group [29]. 

### Cognitive Impairment in Coexisting Hypertension and Chronic Kidney Disease

Cognitive dysfunction in patients with chronic kidney disease (CKD) was the objective of a study conducted by our team. A significant increase in the risk of cognitive dysfunction at each stage of kidney disease was observed. Factors such as low hemoglobin levels (Hb < 11 g/dl), elevated parathyroid hormone (PTH) levels, and a history of diabetes mellitus played a significant role in worsening cognitive function in these patients. Notably, patients with pulse pressure < 60 mmHg or those taking calcium antagonists exhibited better executive function [30]. It was supported that in patients with CKD, high levels of arterial stiffness indices, either directly measured as carotid–femoral pulse wave velocity (cf-PWV) or indirectly estimated as pulse pressure (PP), correlate with established markers of cognitive dysfunction (MMSE) [31]. Furthermore, hypertensive patients with kidney disease receiving renin–angiotensin–aldosterone system (RAAS) inhibition therapy, such as irbesartan, exhibited reduced arterial stiffness indices, improved cognitive function, significant reductions in peripheral and central BP, and decreased white matter hyperintensities [32]. 

Ultimately, uncontrolled hypertension can gradually lead to increased aortic stiffness, which in turn can worsen cognitive function. It is known that hypertension-induced arterial stiffness can cause microcirculation disturbances in the brain, leading to cognitive dysfunction. Another interesting observation is that cognitive dysfunction can itself lead to non-adherence to pharmacological therapy and consequently to uncontrolled hypertension, perpetuating a vicious cycle that may persist for years [33]. Conversely, in hypertensive patients, high adherence to pharmacological treatment not only reduces BP levels but also preserves cognitive and renal function and decreases white matter hyperintensities [32]. 

## 9. Pathophysiology of Hypertension-Induced Cognitive Impairment

**Figure 1:**
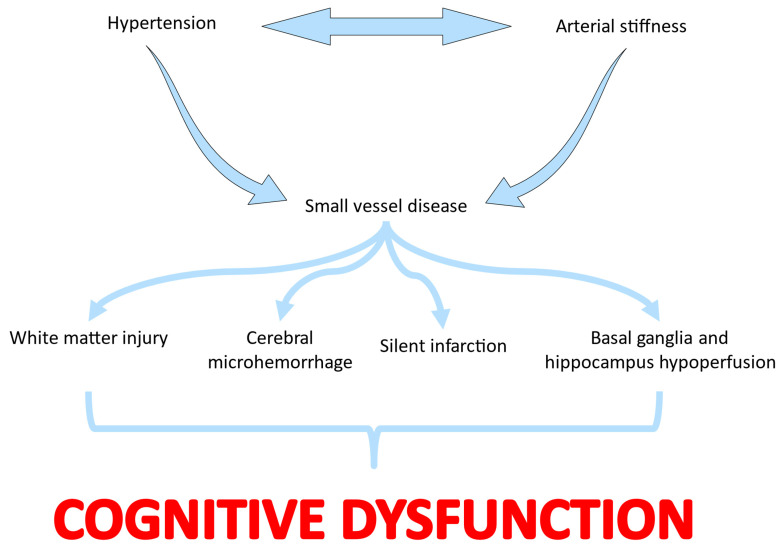
Pathophysiologic mechanisms linking hypertension and arterial stiffness with cognitive dysfunction.

Based on the previously outlined studies and observations, the pathophysiology of cognitive dysfunction in hypertensive patients is associated with the remodeling of small cerebral vessels, which causes increased wall thickness, an increase in the ratio of media thickness to lumen, and a decrease in total wall volume [34]. Additionally, another proposed mechanism suggests that arterial stiffness precedes the onset of hypertension, increasing the likelihood of contributing to its escalation by reducing vascular compliance and increasing pulse pressure [35]. Furthermore, increased arterial stiffness of large arteries and disruption in flow pulsatility contribute to small vessel disease of the brain, resulting in reduced blood flow to specific brain regions associated with cognitive function, such as the basal ganglia and hippocampus [22,36]. Therefore, due to these detrimental microvascular actions, arterial stiffness may serve as a sensitive prognostic factor for subsequent alterations in white matter and cognitive impairment [37]. These small cerebral vessels are unique, and under conditions of uncontrolled hypertension, their cells are subjected to a continuous flow of high-volume blood throughout the cardiac cycle alongside very low vascular resistance. Given the specific anatomy and physiology of these small vessels, the brain tissue is sensitive to microvascular insults and exposure to vascular risk factors. As a result, it is more susceptible to developing what is commonly referred to as small vessel disease of the brain [38,39]. The ultimate consequence of uncontrolled hypertension is subclinical damage to the white matter of the brain, microhemorrhages, and silent infarcts [22] (Figure 1).

## 10. Blood Pressure Control and Improvement in Cognitive Dysfunction

In general, the prevention of cognitive dysfunction primarily involves treating primary diseases and controlling neurological and psychopathological syndromes. However, managing hypertension, atherosclerosis, and heart failure also plays a significant role in improving brain circulation and metabolism. Specifically, the impact of hypertension was studied in the HAAS on Japanese American men, which included systematic monitoring from 1965 [40]. The study assessed the risk of dementia and cognitive dysfunction correlated with the duration of antihypertensive therapy. The sample was grouped by therapy duration (hypertensives never treated, hypertensives treated <5 years, 5 to 12 years, >12 years), with normotensive individuals up to 1991 included as a control group. According to the study results, the longer the duration of antihypertensive treatment, the lower the risk of dementia. The findings indicate that in hypertensive men, the duration of antihypertensive therapy is associated with a reduced risk of dementia and cognitive dysfunction [40]. These results were also confirmed in a more recent meta-analysis of longitudinal cohort studies [7], which showed that hypertensive individuals receiving antihypertensive medications had less cognitive decline over a 20-year follow-up compared to hypertensive individuals not receiving treatment (−0.050 (95% CI, −0.003 to −0.097) vs. −0.079 (95% CI, −0.156 to −0.02 z-score)). The results also suggest that pharmacological therapy may reduce the cognitive decline attributable to hypertension [7].

In a recent post-analysis of patient data from longitudinal studies [41], it was further confirmed that the use of antihypertensive drugs was associated with a reduced risk of developing dementia compared to untreated hypertensive individuals throughout their lives. In the primary, partially adjusted analysis that included 14 studies, untreated hypertensive individuals had a 42% increased risk of dementia compared to healthy controls (hazard ratio (HR), 1.42, 95% CI 1.15–1.76, *p* = 0.001) and a 26% increased risk compared to hypertensive individuals receiving treatment (HR, 1.26; 95% CI, 1.03–1.53, *p* = 0.02). Individuals with hypertension receiving treatment did not have a significantly increased risk of dementia compared to healthy controls (HR, 1.13, 95% CI, 0.99–1.28, *p* = 0.07). The use of antihypertensive drugs was associated with a reduced risk of dementia compared to untreated hypertensive individuals throughout their lives [41].

## 11. Conclusions

Midlife untreated hypertension is associated with worse cognitive function. Aggressive BP lowering and the duration of antihypertensive therapy reduce the incidence of any form of dementia. Reducing the risk of cognitive decline could be one of the multiple targets of antihypertensive therapy, combined with preventing cardiac and chronic kidney disease.

## Data Availability

Not applicable.
